# Utility of Brachial Pulse Pressure and Cerebrospinal Fluid Biomarkers in Assessing White Matter Hyperintensities for Healthy Older Adults

**DOI:** 10.1002/alz70861_108920

**Published:** 2025-12-23

**Authors:** Jeongwoon Kim, Kyoung Shin Park, Ambar Kulshreshtha, Alvaro Alonso, Felicia C. Goldstein, Arshed Quyyumi, James J. Lah

**Affiliations:** ^1^ Emory University School of Medicine, Atlanta, GA USA; ^2^ Emory University Rollins School of Public Health, Atlanta, GA USA; ^3^ Goizueta Alzheimer's Disease Research Center, Emory University, Atlanta, GA USA

## Abstract

**Background:**

Vascular dysfunction has emerged as a potential early contributor to AD/ADRD pathophysiology, highlighting the need for accessible biomarkers to support early detection and interventions. Arterial stiffening may contribute to cerebral microvascular damage and the accumulation of white matter hyperintensities (WMH), a neuroimaging marker associated with AD/ADRD. While cerebrospinal fluid (CSF) biomarkers such as Aβ42/tTau index (ATI) indicate AD/ADRD pathology, the additive value of vascular measures remains unclear. This study investigated whether pulse pressure (PP), a proxy of arterial stiffness, is associated with WMH independently of CSF biomarkers in healthy older adults.

**Methods:**

394 cognitively healthy older adults (age: 62.6±6.6 yrs; 69.7% female; 15.0% African American) from the Emory Healthy Brain Study underwent MRI, lumbar puncture, and blood pressure assessments. PP was calculated as the difference between brachial systolic and diastolic pressure. ATI was calculated from CSF concentrations of Aβ42 and total Tau. Hierarchical linear regression examined the independent contributions of PP and ATI to WMH volume. Covariates (age, sex, education, BMI, race, and mean arterial pressure) were included in step 1, PP and ATI in step 2, and race and age group (older: >62.5, younger: ≤62.5 yrs) interactions in step 3.

**Results:**

Bivariate analyses revealed PP was positively associated with WMH (*r*=0.293, *p*<0.001) while ATI was inversely associated with WMH (*r*=‐0.194, *p*<0.001). After adjusting for covariates, PP was non‐significantly associated with WMH (*β*=0.051, *p*=0.314), while ATI was negatively associated (*β*=‐0.091, *p*=0.031). These associations were modified by age stratification and race. The association between PP and WMH was weaker in younger adults (older: B=0.009, younger: B=0.001, *p*=0.016) and African Americans (African American: B=‐0.016, non‐African American: B=0.001, *p*=0.010). Similarly, the relationship between ATI and WMH was weaker in younger adults (older: B=‐0.187, younger: B=0.056, *p*=0.001) but did not vary by race (*p*=0.794).

**Conclusion:**

Arterial stiffness may complement CSF biomarkers in characterizing WMH burden‐a marker linked to AD/ADRD. The moderating effects of age and race highlight the need for precision approaches in assessing vascular contributions to dementia risk. Given the routine nature of blood pressure assessments, PP may serve as a scalable tool for early risk stratification in aging and dementia research.

